# Food conscientiousness as a buffer against college students' weight gain

**DOI:** 10.3389/fpubh.2025.1434091

**Published:** 2025-03-12

**Authors:** Mitsuru Shimizu, Kimberly Janke, Paul Rose, Jason Murphy

**Affiliations:** Department of Psychology, Southern Illinois University Edwardsville, Edwardsville, IL, United States

**Keywords:** food conscientiousness, conscientiousness, behavioral tendency, weight gain, Freshman 15, college students

## Abstract

**Introduction:**

A variety of psychological factors may influence weight gain among undergraduates. As one of the psychological factors that might influence such weight gain, this research introduces food conscientiousness, a behavioral tendency toward making healthier food choices.

**Methods:**

In Phase 1 of the study, we developed a food conscientiousness scale. In Phase 2, we examined whether undergraduates demonstrated weight gain and whether it was smaller among those high in food conscientiousness.

**Results:**

The results indicated that college students demonstrated weight gain (2 lbs, on average) during the fall 2020 semester. Furthermore, food conscientiousness was significantly negatively associated with weight gain, independent of general conscientiousness. Importantly, this effect was neither moderated by where students lived nor by their perceived access to healthy food, suggesting that food conscientiousness can prevent weight gain regardless of lifestyle.

**Conclusion:**

College undergraduates high (+1 *SD*) in food conscientiousness reported smaller weight gain (0.24 lbs) compared to those low (−1 *SD*) in food conscientiousness (3.93 lbs) during the first 2 months of a fall semester. The results suggest that food conscientiousness may be one of the psychological factors that shapes the extent to young adults gain weight.

## 1 Introduction

### 1.1 Weight gain among college students

Obesity is one of the most significant risk factors for morbidity and mortality (1), affecting all age groups, including people of traditional college age. While the US obesity prevalence was 42.8% in 2017–2018 (2), it is fortunate that obesity prevalence among college students is estimated to be much lower [e.g., 17% in the U.S. according to Stefan et al. (3)]. This may be due in part to students' aversion to weight gain. The idea that students typically gain 15 lbs during their freshman year, the Freshman 15, is commonly feared (4, 5). It should be noted, however, that the empirical evidence for the Freshman 15 has been inconsistent. For instance, because non-college adolescents still gain weight, Baum (6) argued that the Freshman 15 is a myth, and estimated that first-year college students gain only 1 extra lb. on average during their first-year in college [see also (7, 8)]. On the other hand, Holm-Denoma et al. (9) demonstrated that both male and female students gained weight before November of the first academic year (3.4 and 4.0 lbs, respectively), which is much higher than that of average American adults, but much smaller than 15 lbs. By conducting a meta-analysis of 22 published studies on the Freshman 15 effect, Vadeboncoeur et al. (5) also found that about 60% of the first-year undergraduates do gain weight over their first 6 weeks to 8 months, and for those who do gain weight, the average increase is 7.5 lbs. This suggests that the Freshman 15 idea is an exaggeration of a real phenomenon.

Thus, a tendency toward weight gain among university students appears to be real, but folk ideas like the Freshman 15 may exaggerate the extent of such weight gain. However, it is important to recognize that findings of college student weight gain generally reflect *average* weight gain. Mihalopoulos et al. (4) reported that the average weight gain among first-year college students in their study sample was only 2.7 lbs, and while about half of the students gained weight, 15% of them lost weight. Boyce (10) found that weight gain reported by the first-year students was moderated by their original BMI. While those with higher BMIs gained weight, while those with lower BMIs lost weight, especially in the face of stress accompanied with the life transition. Those results, as well as Vadeboncoeur et al.'s (5) finding that 40% of the undergraduates in their meta-analysis did not gain weight, remind us of how much variability there is in whether and how much undergraduate weight changes.

In addition, weight change among college students does not take place only in the first year. For instance, Gropper et al. (11) found that college undergraduates demonstrated an average of 6.61 lbs of weight increase across 4 years. Pope et al. (12) also found such an increase among 86 undergraduates. Their undergraduates gained an average of 9.66 lbs over 4 years and an average of 2.91 lbs in their first year. Notably, in Pope et al.'s study, 20 students were classified as overweight or obese in the first fall semester, while 35 students were classified as overweight or obese in the last spring semester. In addition, among 51 participants classified as healthy weight in the last spring semester, 49 were students who had healthy weight in the first fall semester, but 2 were overweight or obese in the first fall semester. It is important, therefore, to examine what kinds of psychological factors make college students more or less immune to weight gain.

### 1.2 Conscientiousness

One of the most studied personality traits related to physical health is conscientiousness (13), one of the Big Five personality traits. The Big-5 personality dimensions are part of the most widely accepted and researched model of personality [see (14) for reviews]. They consist of Extraversion, Agreeableness, Conscientiousness, Emotional Stability, and Openness. This model of personality traits is hierarchical, consisting of bipolar factors such as Extraversion vs. Introversion, which has lower order facets such as gregariousness and activity. Conscientiousness (as opposed to lack of direction or carelessness) is defined as a relatively stable pattern in the tendency to follow norms and rules, to be goal-directed, and to delay gratification (15). Conscientiousness also has lower order facets, such as dutifulness and self-discipline [e.g., (16)].

As many conscientiousness tendencies, such as delaying gratification and acting organized and efficient, have intuitive connections to healthy behavior, one way in which conscientiousness seems to contribute to physical health is through health-promoting behavior (13). In fact, a meta-analysis involving 194 studies (17) demonstrated that conscientiousness is negatively associated with unhealthy behavior such as drinking and smoking, while it is positively associated with healthy behavior such as exercising. In addition, Gartland et al. (18) found that conscientious people were more likely to adhere to health behavior guidelines such as consuming more fruits and vegetables and reducing drinking and smoking.

However, evidence connecting conscientiousness and weight change is relatively sparse, especially when it comes to weight change during a stressful life adjustment. That is, although many studies have shown an association between conscientiousness and BMI [e.g., (19–21)], none of them have demonstrated that people high in conscientiousness showed less weight gain during a significant life transition compared to people low in conscientiousness. In addition, the majority of participants of those studies were middle-aged adults, not college students. Thus, the first purpose of this study is to examine conscientiousness among undergraduates as a predictor of the weight change. More specifically, we examined whether college students high in conscientiousness showed less weight gain in the fall semester compared to those low in conscientiousness.

### 1.3 Food conscientiousness

We propose that there is a general behavioral tendency that differs among college students that we call food conscientiousness. Students high in food conscientiousness exhibit knowledge, beliefs, attitudes, and motives associated with deliberately choosing and consuming healthier foods. In the terminology of Costa (22), we believe food conscientiousness is a characteristic adaptation rather than a basic trait like general conscientiousness. General conscientiousness might predispose people to engage in behaviors such as healthy eating (23). For example, Keller and Siegrist (24) demonstrated that conscientiousness correlates positively with consumption of healthier food (e.g., fruit) and negatively with consumption of less healthy (e.g., sweet and savory) food [see also (25)]. However, as a characteristic adaptation that can shift in response to changes in basic traits or external influences (22), food conscientiousness may be an even better predictor of healthy eating than general conscientiousness. We conceptualize food conscientiousness as an individual difference variable that includes food-specific knowledge, beliefs, attitudes, and motives. This conceptualization is Whole Trait Theory (26), which highlights the value of analyzing stimulus-specific (e.g., food-specific) cognitive, affective, biological, and motivational processes that influence behavior. While the authors of Whole Trait Theory define traits differently than Costa (22), both perspectives emphasize that behavior (such as eating) can be understood better when cognitive, affective, biological, and motivational personality processes related to a stimulus (such as food) are taken into account.

The second purpose of this study was twofold. We first reported the development and preliminary validation of a new scale of food conscientiousness. After generating a question pool, we conducted exploratory factor analyses to identify and retain the best performing items (Phase 1) in the first sample. We then we verified the unifactorial nature of the scale via factor analyses in the second sample and examined whether the new scale showed good convergent and discriminant validity with existing scales. In particular, we expected to see the significant association between conscientiousness and food conscientiousness. Furthermore, we examined whether the new scale showed good predictive validity such that college students high in food conscientiousness showed reduced susceptibility to weight gain compared to those low in food conscientiousness. We expected to see college students high in food conscientiousness showed less weight gain compared to those low in food conscientiousness above and beyond the effect of conscientiousness (Phase 2).

## 2 Methods

### 2.1 Phase 1

#### 2.1.1 Construction of initial items

We generated 30 one-sentence items based on a scale used by Lee et al. (27), which assessed general shopping habits of consumers such as “I usually read nutrition labels on food products.” In addition, we adopted a scale used by Yahia et al. (28), which was based on the Nutrition Knowledge Questionnaire (29), that assessed how much nutrition knowledge people have to choose healthy food items. Since the main purpose of the study was to determine the effect of a behavioral tendency on weight gain among college students, we avoided items not ecologically valid for undergraduate students such as questions for people diagnosed with chronic diseases. Because behavioral tendencies (22) can consist of behavioral and cognitive components, the first dimension was centered around the behavioral components, with question items such as, “I read the labels on food products while grocery shopping,” while the second dimension was centered around the cognitive components, with items such as, “My nutrition is important to me.”

#### 2.1.2 Final item pool

The items were reviewed by 52 undergraduate students (39 female, 13 male), with a mean age of 20.86 (*SD* = 3.72), recruited from a Health Psychology class the first author taught. Students suggested edits to the wording of questions, as well as removing nine items that were not clearly related to food conscientiousness. For instance, three items were removed because the questions pertained to behavior or cognition outside their control (e.g., “I prepare a majority of the weeks' meals at home”). These changes resulted in a reduction from 30 to 21 items. Later, participants in the primary data collection completed those 21 items using a response scale that ranged from 1 (*disagree very much*) to 7 (*agree very much*).

#### 2.1.3 Participants and procedure

Participants in the primary data collection were 234 undergraduate students (177 female, 57 male) with a mean age of 18.84 (*SD* = 1.17) who received bonus points in Introductory Psychology at a large Midwestern university. Among them, 15.8% identified as Black, 3.0% as Hispanic or Latino, 73.9% as White, 3.0% as Asian, and 4.3% as “other.” During a regular class, participants completed the 21-item food conscientiousness scale developed for this study and demographic questions (i.e., age, gender, ethnicity). The study had Institutional Review Board approval.

#### 2.1.4 Factor structure

A principal components factor analysis to consider possible factor structures for this scale revealed four factors with eigenvalues >1 explained by 64.4% of total variance. However, the scree plots (30) revealed a clear elbow indicating that there was a single factor. In fact, the eigenvalue for the first factor was 9.39 while the eigenvalue for the second factor was markedly smaller at 1.74. While 19 of the 21 items loaded onto the first factor (factor loadings = 0.52–0.82), the other two items cross-loaded weakly and equally on other components. Therefore, we decided to eliminate the two cross-loading items.

Subsequently, another principal components factor analysis for the 19-item scale revealed three factors with eigenvalues >1 explained by the 62.9% of total variance. More importantly, the first factor accounted for the 48.5% of explained variance and all items loaded strongly onto the first factor (factor loadings = 0.53–0.82). Although we expected to see two factor structures corresponding to behavioral and cognitive dimensions of food conscientiousness, this indicated that the proposed food conscientiousness scale is unifactorial. Cronbach's alpha for the 19-item scale showed high internal consistency (α = 0.94). Descriptive statistics for the scale (differentiated by gender) are presented in Table 1.

**Table 1:** Descriptive statistics for food conscientiousness scale.

**Measure**	**Male (*n* = 57)**	**Female (*n* = 177)**	**Total (*n* = 234)**
Mean	3.96	3.77	3.81
SD	0.98	1.20	1.15
Range	1.53–6.32	1.26–7.00	1.26–7.00

### 2.2 Phase 2

Thus, results of Phase 1 suggested good psychometric properties, including good construct validity and high internal consistency of the 19-item food conscientiousness scale. The purpose of Phase 2 was to examine whether this new scale has good convergent and discriminant validity with existing scales. Specifically, we examined whether food conscientiousness was associated with conscientiousness, one of the Big-5 personality dimensions (14), and restrained eating, which reflects how much people are concerned about eating and how easily people are influenced by environmental food cues [e.g., (31)]. We also examined whether food conscientiousness was not associated with other Big-5 personality dimensions such as extraversion. Furthermore, we examined whether college students high in food conscientiousness showed less weight gain during the fall semester compared to those low in food conscientiousness. If our purpose had been to directly examine the Freshman 15 effect, we would have included only first-year students in our sample. However, as previously noted, weight gain can occur across 4 years of college (11, 12), so we did not restrict our sample to first-year students only. It is worth noting, however, that the Introductory Psychology population from which we drew our sample typically consists of about 60% first-year students.

Participants were asked to report their weight in September (Time 1) and November (Time 2) surveys to see whether those who reported higher food conscientiousness demonstrated smaller (or no) weight increases compared to those who reported lower food conscientiousness. Importantly, we expected to see that the predictive effect of food conscientiousness was above and beyond the effects of conscientiousness and dietary restraint. In addition, because weight gain might be especially pronounced among those who lived in dormitories or apartments (instead of at home with their parents, where their eating habits would be less disrupted), we conducted a series of moderated regression analyses to examine whether food conscientiousness could still protect them from weight gain. Alternatively, because weight gain might be especially pronounced among those who do not have access to healthy food (for instance, students who live at home in a community described as a food desert), we also asked participants to report how easily they could access healthy food to examine whether food conscientiousness could help protect them from weight gain.

#### 2.2.1 Participants and procedure

Participants were undergraduates enrolled in Introductory Psychology who were recruited through a participant pool at a large Midwestern university. During the Covid pandemic in the year of 2020, 190 participants completed the first online survey in September and 156 of those participants completed the second online survey in November. On average, participants completed the second survey 50.8 days (37.5 to 64.9 days) after the first survey. Among 156 undergraduate students (122 female, 34 male) with a mean age of 19.61 (*SD* = 3.33), 12.2% identified as Black, 5.8% as Hispanic or Latino, 71.8% as White, 3.8% as Asian, and 6.4% as “other.” Using G^*^Power 3.1 (32), we performed a *post-hoc* sensitivity analysis, which suggested that a sample size of 159 should provide adequate power (0.80) for an effect size of 0.05. Thus, the following results were observed with a very slightly underpowered sample size.

Participants completed measures of food conscientiousness, Big-5 personality traits, and dietary restraint at Time 1. Participants also reported where they lived (1 = home; 2 = dorm; 3 = on-campus housing; 4 = off-campus housing/apartment) at Time 1, and completed a 6-item measure to assess access to healthy food at Time 2 as they moderated the effects of food conscientiousness. In addition, at both Time 1 and Time 2, participants reported demographic (e.g., age, gender, ethnicity) information, weight (to see how much weight they gained), and height (to estimate their BMI). This phase of the study had Institutional Review Board approval.

#### 2.2.2 Measures

##### 2.2.2.1 Food conscientiousness

At Time 1, we measured food conscientiousness by using the scale developed in Phase 1. As in Phase 1, we performed a principal components factor analysis which revealed four factors with eigenvalues >1 that explained 61.9% of total variance. However, scree plots (30) revealed a clear elbow: the eigenvalues for the first and second factors dropped sharply from 7.88 to 1.79, indicating a unifactorial scale. In addition, while 15 of 19 items loaded onto the first factor (factor loadings = 0.50–0.81), four other items cross-loaded weakly and equally on other components. We decided to eliminate these four items.

Consequently, another principal components factor analysis for the 15-item scale revealed that three factors with eigenvalues >1 explained 58.1% of total variance. More importantly, the first factor accounted for 48.8% of the explained variance, and all items loaded strongly onto the first factor (factor loadings = 0.57–0.82). This indicated that the food conscientiousness scale is unifactorial. We simply averaged scores from the 15 items (see Appendix) to create a single index of food conscientiousness. Cronbach's alpha for the 15-item scale showed high internal consistency (α = 0.92).

##### 2.2.2.2 Conscientiousness

At Time 1, to establish convergent and divergent validity for the food conscientiousness scale, participants completed the Ten Item Personality Inventory [TIPI; (33)], which contains two questions for each of the Big-5 personality traits. Participants responded on a scale ranging from 1 (*strongly disagree*) to 7 (*strongly agree*). The two items for Extraversion, Conscientiousness, Emotional Stability, and Openness were significantly correlated with each other, *r* = 0.64, *r* = 0.43, *r* = 0.55, and *r* = 0.18, respectively. However, the two items for Agreeableness were not associated, *p* > *0.9*9. Reliability estimates of the first 4 personality dimensions were α = 0.78, α = 0.60, α = 0.71, and α = 0.31, respectively, suggesting that all personality dimensions except openness and agreeableness could be used for the following analyses. We averaged scores from those three scales such that higher scores indicated higher Extraversion, Conscientiousness, and Emotional Stability.

##### 2.2.2.3 Dietary restraint

At Time 1, we measured participants' concern about eating as it related to their general eating habits with the 10-item Restrained Eating Scale (34). As in previous research [e.g., (31)], we simply averaged scores from the 10 items to create a single index of dietary restraint (α = 0.76) in which higher scores indicated high levels of dietary restraint.

##### 2.2.2.4 Access to healthy food

At Time 2, we measured how easily participants could access healthy food by asking six questions such as “It's easy for me to buy healthy food,” and, “I feel that I am struggling to learn how to prepare healthy food for myself.” After three negative items were reverse coded, we simply added scores from the six items so that higher scores indicated easier access to healthy foods (α = 0.84).

#### 2.2.3 Data analyses

Data were analyzed using SPSS version 29.0 (SPSS, Inc.). Descriptive statistics and intercorrelations were performed for all study variables. We conducted a series of hierarchical regression analyses. In the first analysis, we examined whether weight gain (i.e., weight difference between Time 1 and Time 2) was predicted by the main predictors of this study—conscientiousness and food conscientiousness. In this analysis, gender, BMI, and dietary restraint were included to see if any of those variables covaried with the main predictors. In the second analysis, we examined whether weight gain was predicted by food conscientiousness and where participants lived (dichotomous variable; home = 1, not home = 2), and their interaction term. In the third analysis, we examined whether weight gain was predicted by food conscientiousness and accessibility to health food, and their interaction term. All variables were mean-centered [see (35)].

## 3 Results and discussion

### 3.1 Descriptive statistics and intercorrelations

Table 2 depicts intercorrelations and descriptive statistics. Notably, participants reported greater weight at Time 2 (*M* = 154.33 lbs, *SD* = 31.94) compared to Time 1 (*M* = 152.20 lbs, *SD* = 30.56). The weight difference was statistically significant, *t* = 3.08, *p* = 0.002. Thus, undergraduate students did demonstrate weight gain (2.13 lbs, on average), which was observed in less than 2 months, consistent with previous research that has shown modest weight gain over short academic periods (11, 12). If we assume that those participants were mostly first-year students, these results were consistent with the Freshman 15 effect, although observed weight gain was much smaller than 15 lbs [e.g., (5)].

**Table 2:** Correlations and descriptive statistics.

**Measure**	**1**	**2**	**3**	**4**	**5**	**6**	**7**	**8**	**9**	**10**
1. Gender (male = 1, female = 2)	—									
2. BMI	0.16^*^	—								
3. Food conscientiousness	0.00	−0.07	—							
4. Extraversion	0.15	−0.02	0.06	—						
5. Conscientiousness	0.16^*^	−0.11	0.28^***^	0.11	—					
6. Emotional stability	−0.20^*^	−0.09	0.25^**^	0.19^*^	0.34^***^	—				
7. Restraint eating	0.21^**^	0.33^***^	0.25^**^	0.06	−0.06	−0.20^*^	—			
8. Where participants lived (home = 1, not home = 2)	0.07	0.12	−0.01	0.14	0.01	0.06	0.01	—		
9. Access to healthy food	0.08	−0.04	0.51^***^	0.08	0.30^***^	0.23^**^	−0.00	0.11	—	
10. Difference in weight at Time 1 and 2	0.05	0.05	−0.20^*^	0.08	−0.09	−0.17^*^	−0.09	−0.01	−0.01	—
*M*	1.78	24.78	4.14	7.96	11.34	8.05	3.75	1.53	4.46	2.13
*SD*	0.41	5.03	1.12	3.12	2.19	2.81	1.37	0.50	1.22	8.65

* *p* < 0.05.

** *p* < 0.01.

*** *p* < 0.001.

Furthermore, as shown in Table 2, while food conscientiousness was significantly associated with conscientiousness, *r* = 0.28, *p* < 0.001, it was not associated with extraversion, *p* > 0.43. Thus, the first correlation indicates convergent validity, while the second indicates discriminant validity. However, food conscientiousness was also significantly associated with emotional stability, *r* = 0.25, *p* < 0.01. This result makes sense given that eating often occurs for emotional reasons, and high emotional stability includes better impulse control [e.g., (36)]. Further attesting to convergent validity, food conscientiousness was positively associated with restrained eating *r* = 0.25, *p* < 0.01, suggesting that people who deliberately choose healthier food are more likely to be concerned about their eating.

### 3.2 Primary analysis

For all of the following multiple regression analyses, predictors had good multivariate properties (i.e., VIF scores were <1.34). In the first analysis, we examined whether weight gain was predicted by conscientiousness and food conscientiousness. Only food conscientiousness significantly predicted less weight gain, *B* = −1.55, *t*_(144)_ = −2.21, *p* = 0.03. Because none of the other variables were significant predictors, *p*s > 0.05, they were removed from the following analyses.

The second analysis, which included where participants lived and the interaction between living location and food conscientiousness, revealed that only food conscientiousness significantly predicted less weight gain, *B* = −1.55, *t*_(152)_ = −2.51, *p* = 0.01. Neither where participants lived nor its interaction with food conscientiousness reliably predicted weight change, *p*s > 0.93.

Similarly, the third analysis, which included accessibility to health food and the interaction between this accessibility and food conscientiousness, revealed that only food conscientiousness significantly predicted less weight gain, *B* = −2.05, *t*_(152)_ = −2.87, *p* = 0.005. Neither accessibility to health food nor conscientiousness reliably predicted weight change, *p*s > 0.18.

**Figure 1:**
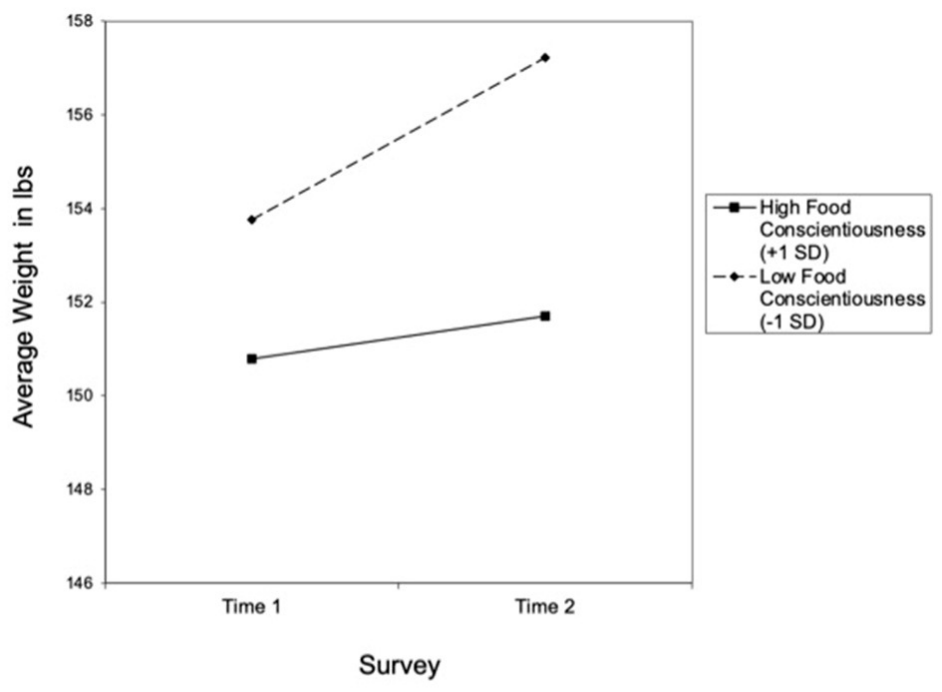
Weight change from Time 1 to Time 2 as a function of food conscientiousness.

Importantly, when the second and third analyses were repeated including general conscientiousness, the same significant results were observed, suggesting that food conscientiousness predicted less weight gain above and beyond the effects of general conscientiousness. Namely, food conscientiousness was negatively associated with weight gain, regardless of where they lived or how accessible they perceived healthy food to be. As indicated in Figure 1, college students high (+1 *SD*) in food conscientiousness reported smaller weight gain (0.24 lbs) compared to those low (−1 *SD*) in food conscientiousness (3.93 lbs).

## 4 General discussion

In this research, we examined whether there is a behavioral tendency to engage in healthy eating and whether students who have this tendency are less likely to gain weight. In Phase 1, we generated items to measure food conscientiousness and refined the scale through exploratory factor analyses. Ecologically valid questionnaire items were selected to assess how much students deliberately choose and consume healthier foods. Although we expected that the scale consisted of behavioral and cognitive components as behavioral tendencies (22), the final factor solution revealed a single factor, which reflects food-specific knowledge, beliefs, attitudes and motives. As such, we argue that food conscientiousness is consistent with more advanced personality theories such as Whole Trait Theory (26).

Furthermore, after determining that the scale is highly reliable, we further validated the scale by examining its discriminant and convergent validity in Phase 2. Importantly, we also found that food conscientiousness was associated with less weight gain among college students. While undergraduate students gained about 2 lbs in 2 months [which, given the nature of our sample, suggests again that “Freshman 15” is an exaggerated term; see (4–6)], those who were high in food conscientiousness exhibited virtually no weight increase. In other words, food conscientiousness acted as a buffer against the weight gain often demonstrated by college students [e.g., (5, 11, 12)]. Thus, this research suggests that food conscientiousness, which encompasses knowledge, beliefs, attitudes, and motives toward healthy eating, guides students to deliberately choose and consume healthier foods.

It is worth noting that the effect of food conscientiousness was independent from the effects of conscientiousness, one of the Big Five personality traits which appears to be associated with healthier lifestyles (17). As a behavioral tendency specific to healthy eating, food conscientiousness predicted less weight gain among undergraduates undergoing a significant life transition. This demonstration of predictive validity is important because conscientiousness has been associated with healthy eating [e.g., (17, 18, 24, 25)] and lower weight [e.g., (19–21)].

In addition, since conscientiousness was not associated with weight gain, food conscientiousness exhibited divergent validity over conscientiousness. Furthermore, the effect of food conscientiousness was observed regardless of where students live and whether they reported access to healthy food, suggesting that food conscientiousness is a behavioral tendency that universally protects college students from weight gain. This research also converges with prior research [e.g., (5)] to suggest that the Freshman 15 effect highlights a phenomenon of concern (i.e., frequent weight gain among young undergraduates); however, it is poorly named. Most undergraduates appear to gain far less than 15 lbs in a year, and some do not gain weight at all.

Because this research did not focus on only first-year students, future studies should examine the effect of food conscientiousness specifically among first-year students (which might suggest that food conscientiousness diminishes the Freshman 15 effect). It would also be helpful to compare the relations between food conscientiousness and weight change between undergraduates and young adults who are not students. Furthermore, it is important to examine if those high in food conscientiousness are more likely to eat healthy food items such as fruit and vegetables, instead of sweets and soft drinks that are often consumed by college students during times of stress (37). Namely, future study should examine how food conscientiousness that encompasses cognitive (e.g., nutritional knowledge) and motivation (to stay healthy) as a behavioral tendency (22) led students to engage in healthy eating.

As a variety of psychological factors that influence eating and obesity among college students are identified (38), future studies should examine how these factors are related to food conscientiousness. For example, food conscientiousness might relate to an increased sense of control and self-efficacy, and a higher-order self-perception, empowerment [see (39)]. These characteristics may work together or independently to reduce eating disturbance. Future studies should also look at the effects of food conscientiousness in the context of intervention techniques designed to control weight. For instance, it may help to examine whether smartphone applications known to facilitate weight loss [e.g., (40)] are most effective among people who are high or low in food conscientiousness. It is possible that such applications allow people low in food conscientiousness to think and behave more like people high in food conscientiousness.

There are three major limitations in this research. First, this research included undergraduate students who were not diverse enough to represent the national college student population. In fact, our sample predominantly consisted of White students (over 70% in both studies), which is disproportionate for American college students in general. In addition, we did not assess their parents' socioeconomic status, which might influence their general eating habits. Second, our cross-sectional design does not allow us to determine any causal relations among the variables in Phase 2. It may be that those who did not exhibit weight gain actually developed greater food conscientiousness, although we assessed food conscientiousness at Time 1, which should make this interpretation less tenable. Finally, because both studies were conducted during the Covid-19 pandemic, our findings might apply only in the situation where students were mostly in quarantine. This means that, although we found that food conscientiousness was related to lower weight gain, this finding might have been observed only because of the tightly controlled physical and social environment during the pandemic.

Nonetheless, it is still noteworthy that this research demonstrated weight gain similar to what has been observed in other studies with U.S. undergraduates, even during the Covid-19 pandemic when students were less likely to engage in social eating, which typically results in overeating [e.g., (41)]. We hope that future research examines whether food conscientiousness prevents weight gain among more diverse college students when pandemic conditions are not present.

## Data Availability

The raw data supporting the conclusions of this article will be made available by the authors, without undue reservation.

## Appendix

1. I read the labels on food products while grocery shopping.
2. I make sure to eat sufficient amounts of vegetables every day.
3. I know my nutrient needs and try to meet them.
4. I spend time learning about nutrition related to my body's needs.
5. I understand nutrition basics and apply this knowledge to my life.
6. I pay attention to the way my body feels after eating different foods.
7. I enjoy eating to benefit my health.
8. I know where my food comes from and how it is made.
9. Where my food comes from is important to me.
10. My nutrition is important to me.
11. I stay educated on current nutrition news.
12. I read about the benefits of different healthy foods.
13. I try to keep a routine when it comes to my meals.
14. When shopping for food, I consider the source of the food products.
15. I try to avoid buying food from large corporate grocery chains.
